# Correlations between cerebrospinal fluid homovanillic acid and dopamine transporter SPECT in degenerative parkinsonian syndromes

**DOI:** 10.1007/s00702-023-02611-y

**Published:** 2023-03-04

**Authors:** Ryoji Goto, Masanori Kurihara, Masashi Kameyama, Hiroki Komatsu, Masashi Higashino, Keiko Hatano, Ryoko Ihara, Mana Higashihara, Yasushi Nishina, Tomoyasu Matsubara, Kazutomi Kanemaru, Yuko Saito, Shigeo Murayama, Atsushi Iwata

**Affiliations:** 1 Department of Neurology, Tokyo Metropolitan Geriatric Hospital and Institute of Gerontology, 35-2 Sakae-cho, Itabashi-ku, Tokyo, 173-0015 Japan; 2 Department of Diagnostic Radiology, Tokyo Metropolitan Geriatric Hospital and Institute of Gerontology, 35-2 Sakae-cho, Itabashi-ku, Tokyo, 173-0015 Japan; 3 Department of Neuropathology (The Brain Bank for Aging Research), Tokyo Metropolitan Geriatric Hospital and Institute of Gerontology, 35-2 Sakae-cho, Itabashi-ku, Tokyo, 173-0015 Japan; 4 Brain Bank for Neurodevelopmental, Neurological and Psychiatric Disorders, United Graduate School of Child Development, Osaka University, 2-2 Yamadaoka, Suita City, Osaka 565-0871 Japan; 5 Present Address: Department of Neurology, The University of Tokyo Hospital, 7-3-1 Hongo, Bunkyo-ku, Tokyo, 113-8655 Japan

**Keywords:** Parkinson’s disease, Progressive supranuclear palsy, Cerebrospinal fluid, Homovanillic acid, Dopamine transporter, SPECT

## Abstract

Both cerebrospinal fluid (CSF) homovanillic acid (HVA) and striatal dopamine transporter (DAT) binding on single-photon emission computed tomography (SPECT) reflect nigrostriatal dopaminergic function, but studies on the relationship between the two have been limited. It is also unknown whether the reported variance in striatal DAT binding among diseases reflects the pathophysiology or characteristics of the subjects. We included 70 patients with Parkinson’s disease (PD), 12 with progressive supranuclear palsy (PSP), 12 with multiple system atrophy, six with corticobasal syndrome, and nine with Alzheimer’s disease as disease control, who underwent both CSF analysis and ^123^I-N-ω-fluoropropyl-2β-carbomethoxy-3β-(4-iodophenyl)nortropane (^123^I-ioflupane) SPECT. We evaluated the correlation between CSF HVA concentration and the specific binding ratio (SBR) of striatal DAT binding. We also compared the SBR for each diagnosis, controlling for CSF HVA concentration. The correlations between the two were significant in patients with PD (*r* = 0.34, *p* = 0.004) and PSP (*r* = 0.77, *p* = 0.004). The mean SBR value was the lowest in patients with PSP and was significantly lower in patients with PSP than in those with PD (*p* = 0.037) after adjusting for CSF HVA concentration. Our study demonstrates that striatal DAT binding correlates with CSF HVA concentration in both PD and PSP, and striatal DAT reduction would be more advanced in PSP than in PD at an equivalent dopamine level. Striatal DAT binding may correlate with dopamine levels in the brain. The pathophysiology of each diagnosis may explain this difference.

**Supplementary Information:**

The online version contains supplementary material available at 10.1007/s00702-023-02611-y.

## Introduction

Degenerative parkinsonian syndromes include idiopathic Parkinson’s disease (PD), progressive supranuclear palsy (PSP), multiple system atrophy (MSA), and corticobasal syndrome (CBS). Although the clinical course and neurological findings are indispensable for disease monitoring, biomarkers that reflect the underlying pathophysiology are important to further understand these diseases.

Homovanillic acid (HVA) is a major metabolite of dopamine that is metabolized by monoamine oxidase and catechol-O-methyltransferase (Mena et al. 1984). Since previous studies have shown that the cerebrospinal fluid (CSF) concentration of HVA is decreased in patients with untreated PD, increases after the introduction of levodopa therapy, and correlates with motor impairment in patients with PD, it is generally considered that low CSF HVA concentration reflects the depletion of dopamine in the brain, mainly in the nigrostriatal system (Jiménez-Jiménez et al. 2014; Kremer et al. 2021; Mena et al. 1984; Stefani et al. 2017). Although a previous multicenter study questioned the utility of its measurement as a disease progression marker (Parkinson Study Group 1995), a recent single-center study using a better measurement protocol suggested its utility in evaluating nigrostriatal pathway damage (Kremer et al. 2021). CSF HVA measurement can also be useful in parkinsonian syndromes besides PD (Duvoisin et al. 1987).

The dopamine transporter (DAT) is a transmembrane protein that regulates the reuptake of dopamine, located at the presynaptic terminal of the dopaminergic neuron axon in the striatum (Booij et al. 1997; Palermo and Ceravolo 2019). Striatal DAT binding on DAT single-photon emission computed tomography (SPECT) has been proposed to represent the amount of DAT (Booij et al. 1997; Palermo and Ceravolo 2019). While it is widely used to assess presynaptic dopaminergic deficits in the diagnosis of parkinsonian syndromes (Booij et al. 1997), it remains to be known what the quantitative measurement of striatal DAT binding intensity reflects (Brücke and Brücke 2022). Although several autopsy studies have reported that antemortem striatal DAT binding is proportional to the number of nigral dopaminergic cells at autopsy (Snow et al. 1993; Colloby et al. 2012; Kraemmer et al. 2014), others have reported conflicting results (Saari et al. 2017; Honkanen et al. 2019). Studies using autopsy brains may have some limitations, including the time interval and effect of dopaminergic treatment.

As these examinations are both considered to be related to dopaminergic deficits in the central nervous system, mainly in the nigrostriatal system (Mena et al. 1984), the results of these two tests would correlate with each other. However, the association between the two has not been investigated among parkinsonian syndromes other than PD. A demonstration of this correlation would clarify the clinical significance of striatal DAT binding. Moreover, although striatal DAT binding has been previously reported to be lower in PSP than in PD and MSA (Badoud et al. 2016; Kaasinen et al. 2019), it is unknown whether the difference reflects the pathophysiological differences between diseases, since disease duration and severity were not consistent among the diagnoses in these studies. Comparing the correlations between each diagnosis would be useful in determining the cause of the difference.

In this study, we aimed to elucidate the clinical significance of DAT SPECT by examining the association between CSF HVA concentration and striatal DAT binding in each parkinsonian syndrome and discuss the differences in the pathophysiology by comparing them among each diagnosis after adjustment.

## Materials and methods

### Patients

This study was approved by the Institutional Review Board of the Tokyo Metropolitan Institute of Gerontology and was performed in accordance with the ethical standards of the 1964 Declaration of Helsinki and its later amendments. Written informed consent was obtained from all participants or their caregivers. In this study, we retrospectively recruited patients who were diagnosed with parkinsonian syndromes, or Alzheimer’s disease (AD) as disease controls, and underwent both CSF HVA measurement and ^123^I-ioflupane SPECT at our hospital between March 2014 and June 2022. The interval between the two tests was < 3 months. We excluded patients who had other known neurological comorbidities or had ever been on medications that could affect the test results, such as anti-parkinsonian drugs, sulpiride, or mirtazapine (Kadoguchi et al. 2014) before HVA measurement and those drugs previously reported to affect DAT SPECT results (Kägi et al. 2010). Patients with clinical diagnoses of PD fulfilling the UK Parkinson’s Disease Society Brain Bank clinical diagnostic criteria (Hughes et al. 1992), probable PSP according to the 2017 MDS clinical diagnostic criteria (Höglinger et al. 2017), probable MSA according to the second consensus criteria (Gilman et al. 2008), CBS fulfilling the modified Cambridge criteria (Mathew et al. 2012), and AD dementia confirmed as A + T + N + by CSF biomarkers according to the 2018 National Institute on Aging-Alzheimer’s Association research framework (Jack et al. 2018) were included. Clinical data, including age, sex, past medical history, presenting symptoms, clinical course, medications, neurological findings including the Geriatric Depression Scale (GDS), and cognitive function tests such as the Mini-Mental State Examination (MMSE) and Frontal Assessment Battery (FAB) at the time CSF analysis was performed, were retrospectively collected for all patients.

### CSF analysis

CSF was obtained via lumbar puncture in all patients. The first 3 mL of CSF was used for cell counting and routine biochemical testing, and an additional 2 mL was collected directly in polypropylene tubes. CSF HVA concentration was measured using a high-performance liquid chromatography system equipped with electrochemical sensors, as previously described (Morimoto et al. 2017).

### SPECT imaging

DAT SPECT images were acquired 3 h after intravenous administration of 185 MBq of ^123^I-N-ω-fluoropropyl-2β-carbomethoxy-3β-(4-iodophenyl)nortropane (^123^I-ioflupane, DaT SCAN®, Nihon Medi-Physics) using a gamma camera Infinia Hawkeye 4 (GE Healthcare, Milwaukee, WI) equipped with an extended low-energy general-purpose collimator. Imaging parameters were as follows: matrix size, 128 × 128; pixel size, 3.22 mm; and energy window, 159 keV ± 10%. Data were acquired for 34 min and reconstructed using an ordered subset expectation maximization method (iteration, 6; subset, 10) on a Xeleris workstation (GE Healthcare) without attenuation correction or scatter correction.

The specific binding ratio (SBR) of the striatal DAT binding was semi-quantitatively calculated with DAT VIEW software (Nihon Medi-Physics, Tokyo, Japan) using the Southampton method (Tossici-Bolt et al. 2006) after phantom calibration, according to a previously reported method (Matsuda et al. 2018). The mean of the left and right SBRs was used to evaluate the association in the analyses. The asymmetry index (A.I.) of the SBR was calculated using software.

### Statistics

Categorical variables were compared using the *χ*^2^ test. Normally distributed continuous variables were expressed as mean ± standard deviation and were compared using analysis of variance, while continuous variables without a normal distribution were expressed as median (interquartile range) and were compared using the Kruskal–Wallis test among each diagnosis. Pearson’s correlation test was used to verify the correlation between HVA concentration and SBR. In disease groups for which a significant correlation between the two was identified, we also conducted a one-way analysis of covariance (ANCOVA) to evaluate the differences in SBRs among each diagnosis, controlling for CSF HVA concentrations. The estimated SBR values when the CSF HVA concentrations were aligned to the overall average were compared for each diagnosis. Statistical significance was set at *p* < 0.05 and was adjusted according to the Bonferroni correction for multiple comparisons.

All statistical analyses were performed using R version 4.2.1 (R Foundation for Statistical Computing, Vienna, Austria).

## Results

### Clinical features of the patient groups

A total of 74 patients with PD, 13 with PSP, 12 with MSA, six with CBS, and nine with AD as disease controls fulfilled the inclusion criteria. Four patients with PD and one with PSP were excluded from the analyses because of comorbidities. The MSA patients consisted of five with MSA-P and seven with MSA-C. MMSE scores were recorded in 56 patients with PD, 11 with MSA, and all patients with PSP, CBS, or AD. FAB was recorded in all patients whose MMSE scores were recorded, except for one patient with PD and one with AD. The GDS was recorded in 54 patients with PD, 11 with MSA, eight with PSP, four with CBS, and five with AD. Two patients with PSP were conducted autopsy. In both cases, intracerebral aggregation of four repeat-tau neurofibrillary tangles and tufted astrocytes was observed, and consistent with both decreased CSF HVA concentration and low striatal DAT binding, melanin-containing neurons were depleted in the substantia nigra (Table S1 and Fig. S1 provided as Online Resource).

Patient characteristics are presented in Table 1. No significant difference was observed among the diagnoses regarding age, sex, duration of symptoms, interval period, or GDS. The mean Hoehn–Yahr score was lower in patients with PD than in those with PSP (*p* = 0.006, after p-value correction for multiple comparisons). The mean MMSE score was higher in patients with PD than in patients with PSP (*p* < 0.001), CBS (*p* = 0.003), and AD (*p* < 0.001), and was lower in patients with PSP than in those with MSA (*p* < 0.001). The A.I. was also significantly lower in patients with PD than in those with PSP (*p* = 0.011), and in AD than in those with PSP (*p* = 0.002).

**Table 1:** Characteristics of patient groups

	PD (*n* = 70)	PSP (*n* = 12)	MSA (*n* = 12)	MSA-P (*n* = 5)	MSA-C (*n* = 7)	CBS (*n* = 6)	AD (*n* = 9)	*P* value
Age	75.7 ± 7.7	77.2 ± 4.2	70.9 ± 10.4	74.0 ± 7.8	68.7 ± 11.4	72.3 ± 8.8	79.9 ± 8.6	0.101^a^
No (%) of women	36 (51)	6 (50)	7 (58)	3 (60)	4 (57)	2 (33)	6 (67)	0.127^c^
Duration of symptoms, years	1.7 ± 1.8	2.1 ± 1.3	2.0 ± 2.1	1.5 ± 1.3	2.5 ± 2.4	2.1 ± 1.9	–	0.825^a^
Interval period between CSF analysis and DAT SPECT, days	21.3 ± 14.0	22.3 ± 18.7	17.2 ± 12.7	12.4 ± 10.6	20.6 ± 13.0	29.3 ± 15.7	30.8 ± 27.4	0.628^a^
Hoehn–Yahr score	3 (2–4)	3 (3–4)	3 (2.8–3)	3 (3–3)	3 (2–3)	3 (2.3–3)	–	0.035^b^
MMSE	28 (26–29)	21 (10–23)	25 (23–27.5)	25 (25–27)	27 (24.8–27.8)	18.5 (16–24)	20 (19–23)	< 0.001^b^
FAB	14.5 (13–16)	9 (4–11.5)	12 (11.5–13.5)	13 (13–14)	11.5 (11–12)	8 (5–15)	10 (9–12)	< 0.001^b^
GDS	4 (1.25–7)	3 (2–4.25)	5 (3–10)	8 (5–12)	4 (3–5)	6 (2.75–10)	3.5 (0.5–6.5)	0.173^a^
CSF HVA, ng/mL	24.0 ± 14.0	26.6 ± 15.9	21.9 ± 14.1	16.9 ± 7.6	25.5 ± 16.4	26.7 ± 6.9	46.9 ± 14.6	< 0.001^a^
Average SBR	2.41 ± 1.43	1.53 ± 1.38	3.11 ± 0.74	2.76 ± 0.53	3.35 ± 0.77	2.46 ± 0.73	4.77 ± 0.83	< 0.001^a^
A.I	19.1 (7.2–35.7)	55.1 (31.1–94.4)	26.8 (9.7–37.7)	42.1 (36.2–47.1)	11.6 (2.9–23.7)	32.5 (16.1–43.4)	5.7 (2.4–10.8)	< 0.001^b^

Values are expressed as counts (%), means ± SD (ranges), or medians (interquartile ranges)

*PD* Parkinson’s disease, *PSP* progressive supranuclear palsy, *MSA* multiple system atrophy, *CBS* corticobasal syndrome, *AD* Alzheimer’s disease, *A.I.* asymmetry index, *CSF* cerebrospinal fluid, *DAT* dopamine transporter, *SPECT* single-photon emission computed tomography, *MMSE* mini-mental state examination, *FAB* frontal assessment battery, *GDS* geriatric depression scale, *HVA* homovanillic acid, *SBR* specific binding ratio, *SPECT* single-photon emission computed tomography

*P* values represent the result of ^a^analysis of variance

^b^Kruskal–Wallis, and

^c^*χ*^2^ test

### Correlations between CSF HVA concentration and SBR of striatal DAT binding

Compared with patients with AD, CSF HVA concentration was significantly lower in those with PD (*p* < 0.001), PSP (*p* = 0.017), or MSA (*p* = 0.001), although the differences among each parkinson syndrome were relatively small. The mean SBR was significantly lower in patients with PD (*p* < 0.001), PSP (*p* < 0.001), or CBS (*p* = 0.013) than in AD, and it was significantly lower in PSP than in MSA (*p* = 0.043), even after correction for multiple comparisons.

**Fig. 1:**
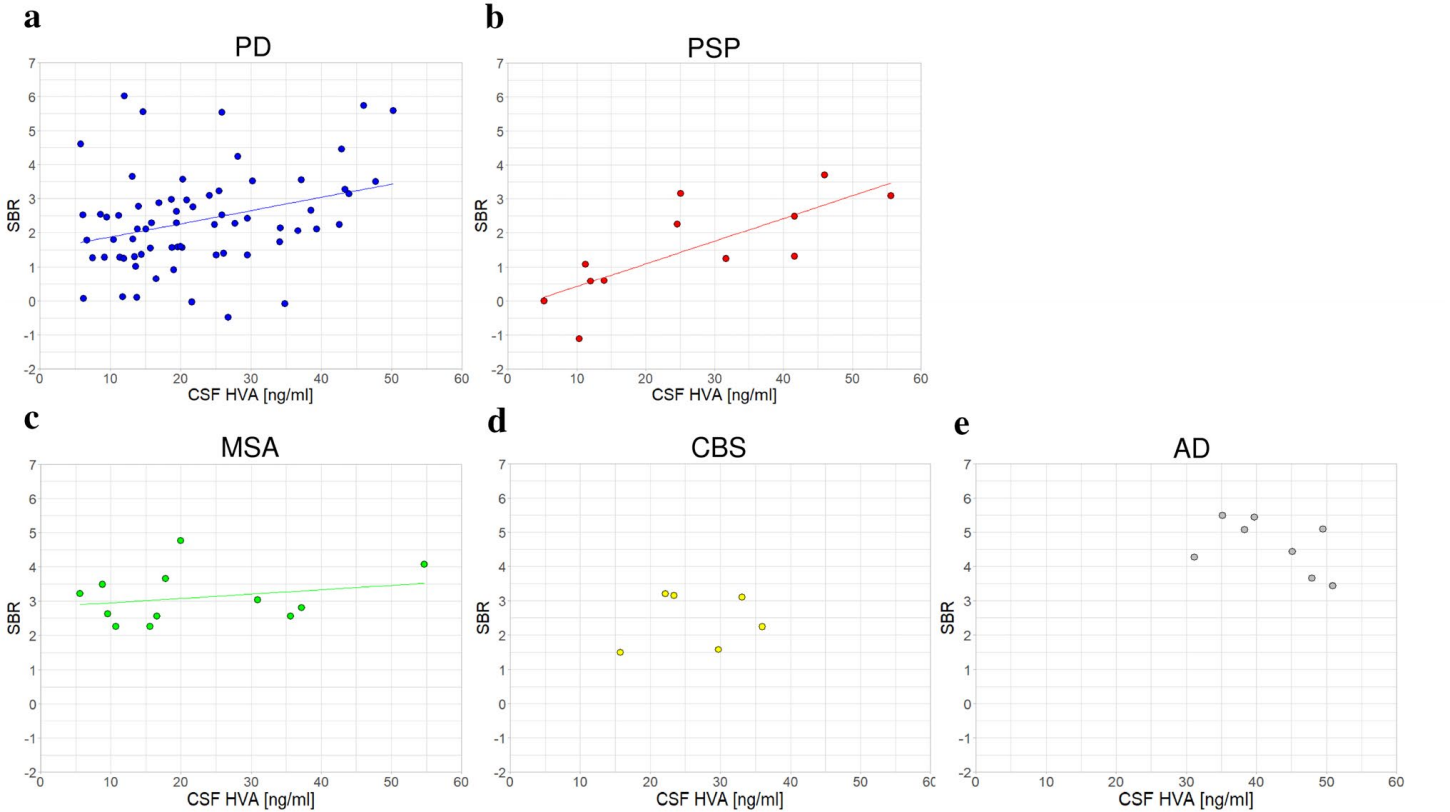
Distributions of CSF HVA concentrations and calibrated average SBRs from DAT SPECT in each diagnosis: PD **a** PSP **b** MSA **c** CBS **d** and AD as disease controls (**e**). A scatter plot is shown with the regression lines for each diagnosis. Significant correlations were observed between the patients with PD (*r* = 0.34, *p* = 0.004) and PSP (*r* = 0.77, *p* = 0.004). *AD* Alzheimer’s disease, *CBS* corticobasal syndrome, *CSF* cerebrospinal fluid, *DAT* dopamine transporter, *HVA* homovanillic acid, *MSA* multiple system atrophy, *PD* Parkinson’s disease, *PSP* progressive supranuclear palsy, *SBR* specific binding ratio, *SPECT* single-photon emission computed tomography

**Fig. 2:**
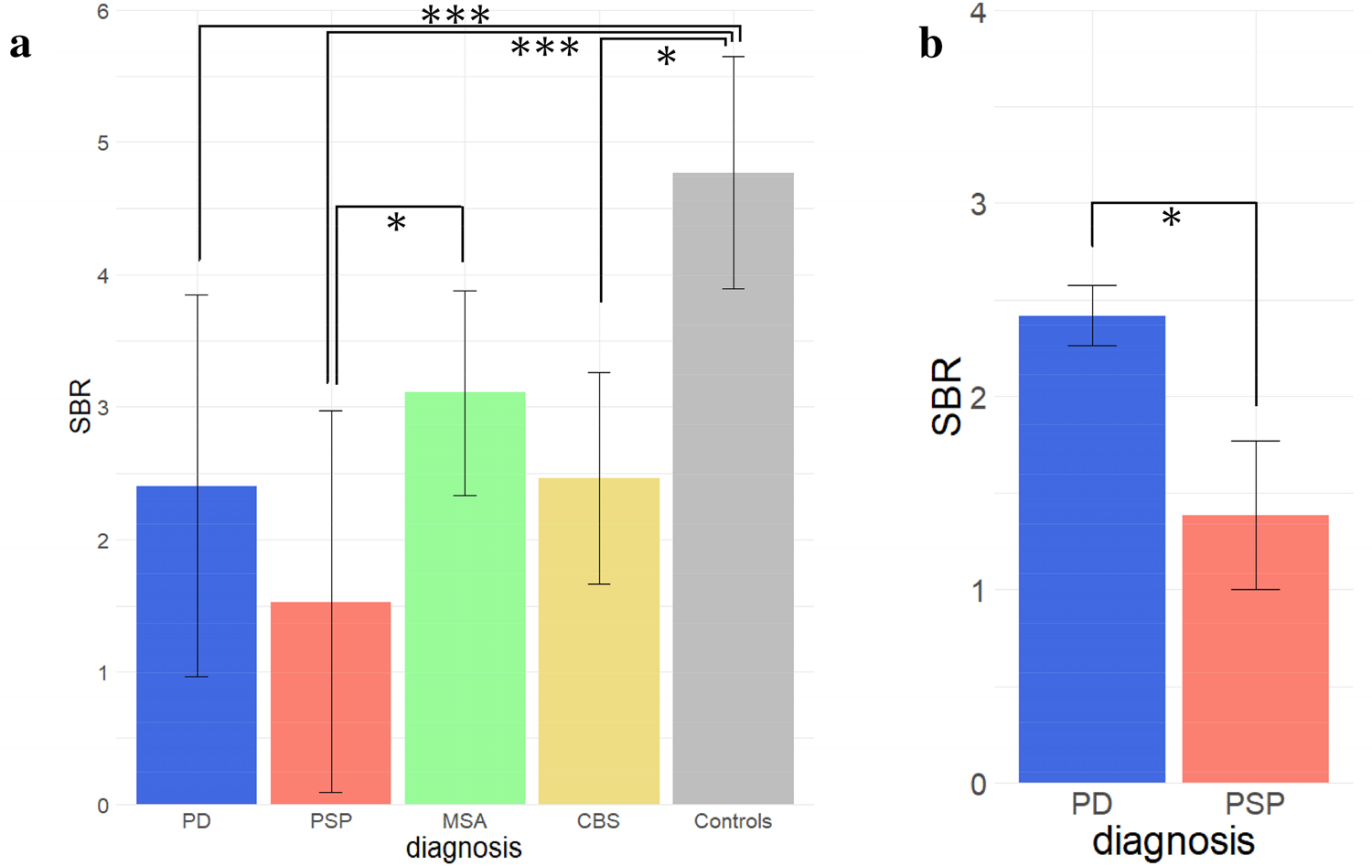
Bar graphs displaying the distribution of SBR in each diagnosis. **a** The mean values of SBR in each diagnosis are shown as bar charts. Standard deviations are shown as error bars. Compared with patients with AD, SBR was significantly lower in patients with PD (*p* < 0.001), PSP (*p* < 0.001), or CBS (*p* = 0.013), and it was significantly lower in PSP than in MSA (*p* = 0.043), after *p*-value correction for multiple comparisons. **b** The estimated values of SBR in PD and PSP, for which a significant correlation was identified, when CSF HVA concentrations were aligned to the overall average by analysis of covariance with CSF HVA concentrations as covariates are shown as bar charts. The SBR was significantly lower in patients with PSP than in those with PD (*p* = 0.037). The error bars indicate a standard error. *p < 0.05, ***p < 0.001. *CBS* corticobasal syndrome, *CSF* cerebrospinal fluid, *HVA* homovanillic acid, *MSA* multiple system atrophy, *PD* Parkinson’s disease, *PSP* progressive supranuclear palsy, *SBR* specific binding ratio

The association between the CSF HVA concentration and SBR was examined for each diagnosis. The correlation was significant in patients with PD (*r* = 0.34, *p* = 0.004) and PSP (*r* = 0.77, *p* = 0.004) but not in those with MSA (*r* = 0.24, *p* = 0.45), CBS (*r* = 0.15, *p* = 0.78), or AD (*r* = 0.33, *p* = 0.40). The scatter plot of patients with regression lines is shown in Fig. 1, and the distribution of SBR for each diagnosis is shown in Fig. 2a.

### Comparison of SBR among each diagnosis

We also compared striatal DAT binding after controlling for the CSF HVA concentration between PD and PSP, for which a significant correlation was identified. After confirming that the interaction effect was not significant (*p* = 0.24), we conducted an ANCOVA with CSF HVA concentrations as covariates. In the analysis, the SBR was significantly lower in patients with PSP than in those with PD (*p* = 0.037). Bar charts considering the covariates are shown in Fig. 2b.

## Discussion

A significant correlation between CSF HVA concentration and SBR of striatal DAT binding was observed not only in patients with PD, but also in those with PSP. Furthermore, striatal DAT binding was lower in PSP than in those with PD, after adjusting for CSF HVA concentration.

The correlation between CSF HVA concentration and striatal DAT binding in patients with PD and PSP indicates the clinical significance of DAT SPECT. The number of previous studies comparing CSF HVA concentrations and striatal DAT binding is scarce, and these studies were limited to patients with PD. Ishibashi et al. (2010) found a significant correlation (*r* = 0.76, *p* < 0.01) between CSF HVA concentrations and the striatal uptake of ^11^C-2β-carbomethoxy-3β-(4-fluorophenyl) tropane positron emission tomography in drug naïve PD patients (*n* = 21). Kremer et al. (2021) reported CSF HVA/dopamine correlating with ^123^I-ioflupane SPECT uptake ratios of the mean caudate (*r* = 0.28, *p* < 0.01) and ipsilateral caudate nucleus (*r* = 0.29, *p* < 0.01) in patients with PD (*n* = 95). As for the comparison of dopaminergic imaging with neuropathology findings, several autopsy studies have reported correlations between striatal DAT binding and the number of nigral dopaminergic cells (Snow et al. 1993; Colloby et al. 2012; Kraemmer et al. 2014). On the other hand, in studies limited to patients with parkinsonian syndromes, no correlation was found between striatal DAT binding and substantia nigra cell counts (Saari et al. 2017) or striatal dopaminergic axons (Honkanen et al. 2019) at autopsy. In a study in monkeys with a one-sided 1-methyl-4-phenyl-1,2,3,6-tetrahydropyridine (MPTP) lesion, there was also no link between striatal DAT binding and cell density of dopaminergic neurons in the substantia nigra when the cell loss exceeded 50%, while the uptake correlated with striatal dopamine levels throughout the full range of dopaminergic cell loss (Karimi et al. 2013). Since a low CSF HVA concentration has been proposed to reflect the depletion of dopamine in the brain, the results of our study suggest that striatal DAT binding correlates with dopamine levels in the brain both in PD and PSP, although further research is required to elucidate the mechanism.

The lower SBR in PSP than in PD after adjusting for CSF HVA concentration may be attributable to the difference in pathophysiology. Several previous studies have reported that striatal DAT binding is lower in patients with PSP than in those with PD and MSA (Badoud et al. 2016; Kaasinen et al. 2019). However, since the baseline characteristics, including disease duration and severity, were not consistent among disease groups in these studies, it was uncertain whether the differences reflect pathophysiological differences between diseases. Although there were no differences in baseline characteristics such as age, sex, or duration of symptoms in our study, it was difficult to match disease severity because of the different nature of the diseases. Therefore, we compared striatal DAT binding among each diagnosis after controlling for CSF HVA concentration. Our results showed that striatal DAT binding was lower in PSP patients than in PD patients, even after adjusting for CSF HVA values. Since all patients underwent CSF collection before the initiation of dopaminergic treatment and the intervals between the two tests were short within 3 months, it is unlikely that the medication or the progression during the intervals influenced the results. These results suggest that striatal DAT reduction is more advanced in patients with PSP than in those with PD at equivalent dopamine levels in the brain. The differences in the pathophysiology of each diagnosis may underlie the discrepancy in striatal DAT binding. As DAT is a protein expressed in the dopaminergic neuron terminals in the striatum, several factors have been assumed to contribute to striatal DAT reduction, including dopaminergic cell loss, axonal dysfunction (Cheng et al. 2010; Kneynsberg et al. 2017; Koziorowski et al. 2021; Schirinzi et al. 2016), and compensatory downregulation of DAT at the presynaptic terminal (Lee et al. 2000; Palermo and Ceravolo 2019). The relative reduction in striatal DAT in PSP may be due to the relative severity of axonal dysfunction or downregulation of DAT at the presynaptic terminal, although we cannot rule out the possibility that CSF HVA values are increased due to differences in the dopamine turnover (Kish et al. 1985; Palermo and Ceravolo 2019), reduction in delivery from the brain into the CSF (Rapoport et al. 2004), or dopamine levels outside the nigrostriatal pathway (Ruberg et al. 1985).

This study had several limitations. First, the number of cases was rather limited, especially for atypical parkinsonian syndromes. The correlations could have been significant if evaluated in a larger number of patients with MSA and CBS, considering the variability in each test result. Second, the diagnoses were based on clinical criteria rather than neuropathological examinations. Only two cases with PSP were performed the autopsy in this study. Although studies with more cases with neuropathologically confirmed diagnoses and comparing those findings would be desirable, since the diagnostic criteria have been well-established and most patients have been followed up by neurologists to review their diagnoses, we presume that the possibility of misdiagnosis would be minimal. Moreover, the Southampton method, which we used in this study for the analysis of the striatal DAT binding, does not allow the analysis by separation of striatal subregions. It has been reported that the striatal DAT binding is lower in patients with PSP than in those with PD or MSA-P, especially in the caudate (Chen et al. 2022; Kaasinen et al. 2019). Future studies should consider evaluating the striatal DAT binding of each subregion separately and comparing the differences among each diagnosis.

Our study showed that striatal DAT binding correlated with the CSF concentration of HVA, a major metabolite of dopamine, in both PD and PSP, and that striatal DAT binding in PSP was significantly lower than that in PD, adjusting for CSF HVA concentration. Both CSF HVA and striatal DAT binding are useful ancillary markers for assessing dopamine levels in the brains of patients with PD and PSP. Further discovery could be expected by comparing DAT SPECT findings with CSF biomarkers, such as HVA.

## Supplementary Information

Below is the link to the electronic supplementary material.

Supplementary file1 Fig. S1 Neuropathological findings. Both cases displayed moderate to severe loss of pigmentation in the substantia nigra (a, e), where globose-shaped neurofibrillary tangles were detected by Hematoxylin Eosin staining (b, f) and immunostaining for four repeat-tau (c, g). Tufted astrocytes were present in the midbrain tegmentum (d) and the putamen (h). These findings support the diagnosis of progressive supranuclear palsy. Scale bars: 1 cm (a, e), 20 μm (b-d, f-h) (DOCX 1672 KB)

## Data Availability

The data supporting the findings of this study are available from the corresponding author upon reasonable request.
