# Supplementary material for: Correlations between cerebrospinal fluid homovanillic acid and dopamine transporter SPECT in degenerative parkinsonian syndromes
Source: J Neural Transm (Vienna). 2023 Mar 4;130(4):513–20. doi: 10.1007/s00702-023-02611-y (PMC10050014; doi:10.1007/s00702-023-02611-y)

**Table S1. Clinical information of autopsied patients**

|  | PSP 04 | PSP 06 |
| --- | --- | --- |
|  |  |  |
| Age at the time of DAT SPECT imaging | 83 | 80 |
| Sex | Female | Male |
| Duration of symptoms,  years | 4 | 4 |
| Interval period between CSF analysis and DAT SPECT,  days | 21 | 17 |
| Interval period between DAT SPECT and autopsy | 15 days | 2 years |
| Hoehn–Yahr score | 5 | 4 |
| CSF HVA, ng/mL | 10.4 | 14.0 |
| Average SBR | -1.12 | 0.60 |

Abbreviations: PSP, progressive supranuclear palsy; DAT, dopamine transporter; SPECT, single-photon emission computed tomography; CSF, cerebrospinal fluid; HVA, homovanillic acid; SBR, specific binding ratio.


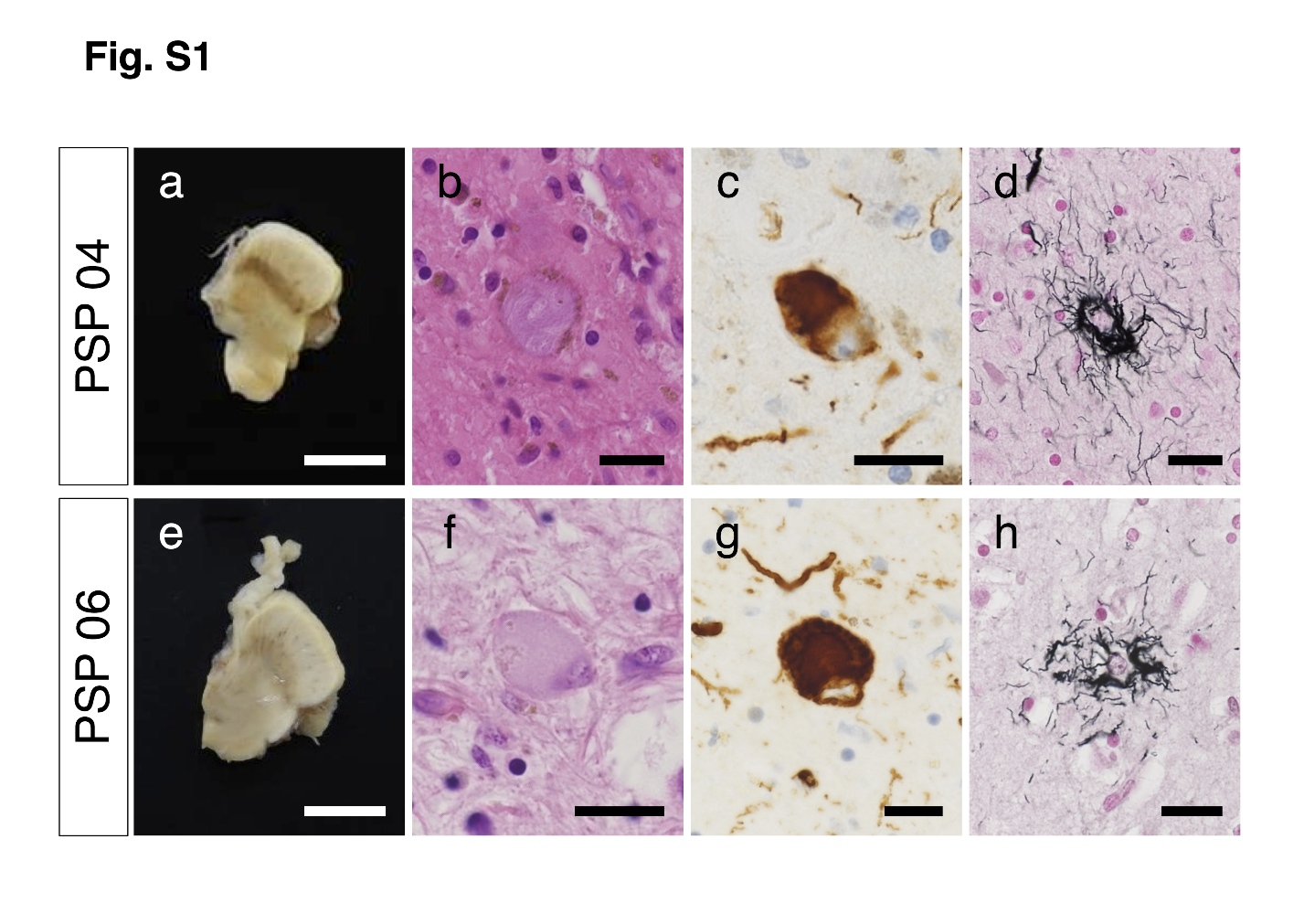

Supplement: Supplementary file 1 — Supplementary file1 Fig. S1 Neuropathological findings. Both cases displayed moderate to severe loss of pigmentation in the substantia nigra (a, e), where globose-shaped neurofibrillary tangles were detected by Hematoxylin Eosin staining (b, f) and immunostaining for four repeat-tau (c, g). Tufted astrocytes were present in the midbrain tegmentum (d) and the putamen (h). These findings support the diagnosis of progressive supranuclear palsy. Scale bars: 1 cm (a, e), 20 μm (b-d, f-h) (DOCX 1672 KB) [file 702_2023_2611_MOESM1_ESM.docx]
